# Emetics

**Published:** 1892-06

**Authors:** 


### EMETICS.

This was a favorable class of remedies with the early eclectics from which they scored many a victory and relieved many patients. Notwithstanding the great tendency towards supportive remedies and measures in medicine, to the exclusion of all depressives, still I think emetics are a class of remedies that do not receive the attention now that they deserve. We are too apt in our haste and eagerness for new remedies and classes to neglect some of the true and tried pearls of great price.

How many times have we seen a bilious fever aborted by a free emesis, a jalap purge and a few doses of quinine? How many cases of dyspepsia might be cured by a thorough stomach cleansing every morning, an hour before breakfast, conjoined with a little liver medicine two or three times a week? Notice how quickly and nicely a tediously labor terminates after nauseating, or even vomiting, your patient with a little lobelia or ipecac. The danger of hemorrhage is not half so great, and placenta more easily and quickly expelled.

One of the best remedies for sick headache is a nice free lobelia or ipecac emesis, followed by $\frac{1}{10}$ gr. pellets of podophyllin and sodibicarb. Even specific medicationists frequently, when an emetic is indicated, neglect it and give the indicated sedative and cathartic. In consequence, they do not get the right answer to their remedies. You all have noticed how nicely and quickly your medicines influence your patient for good, after thoroughly emptying the stomach of all undigested food and other offending matter. It sedates the nervous system and stimulates a free action of the glandular organs, and promotes a free transpiration from the skin.

In children who have convulsions from hard, full stomach, bad breath from undigested food, and constipation, are more quickly relieved by a lobelia emetic than from bromides and chloral. In la grippe, where you have nausea, headache, constipation, offensive breath, backache, soreness of the bowels and dry skin, the usual treatment will act much better by giving a mild ipecac emetic.—*American Med. Journal.*

